# Histological Correlates of Neuroanatomical Changes in a Rat Model of Levodopa-Induced Dyskinesia Based on Voxel-Based Morphometry

**DOI:** 10.3389/fnagi.2021.759934

**Published:** 2021-10-28

**Authors:** Xiaoqian Zhang, Wei Chen, Yi Wu, Weiqi Zeng, Yuhao Yuan, Chi Cheng, Xiaoman Yang, Jialing Wang, Xiaomei Yang, Yu Xu, Hao Lei, Xuebing Cao, Yan Xu

**Affiliations:** ^1^Department of Neurology, Union Hospital, Tongji Medical College, Huazhong University of Science and Technology, Wuhan, China; ^2^State Key Laboratory of Magnetic Resonance and Atomic and Molecular Physics, Innovation Academy for Precision Measurement Science and Technology, National Center for Magnetic Resonance in Wuhan, Chinese Academy of Sciences, Wuhan, China; ^3^University of Chinese Academy of Sciences, Beijing, China

**Keywords:** Parkinson’s disease, L-DOPA-induced dyskinesia, magnetic resonance imaging, voxel-based morphometry, striatum, astrocyte, synaptic plasticity, microvasculature

## Abstract

Long-term therapy with levodopa (L-DOPA) in patients with Parkinson’s disease (PD) often triggers motor complications termed as L-DOPA-induced dyskinesia (LID). However, few studies have explored the pathogenesis of LID from the perspective of neuroanatomy. This study aimed to investigate macroscopic structural changes in a rat model of LID and the underlying histological mechanisms. First, we established the hemiparkinsonism rat model through stereotaxic injection of 6-hydroxydopamine (6-OHDA) into the right medial forebrain bundle, followed by administration of saline (PD) or L-DOPA to induce LID. Magnetic resonance imaging (MRI) and behavioral evaluations were performed at different time points. Histological analysis was conducted to assess the correlations between MRI signal changes and cellular contributors. Voxel-based morphometry (VBM) analysis revealed progressive bilateral volume reduction in the cortical and subcortical areas in PD rats compared with the sham rats. These changes were partially reversed by chronic L-DOPA administration; moreover, there was a significant volume increase mainly in the dorsolateral striatum, substantia nigra, and piriform cortex of the lesioned side compared with that of PD rats. At the striatal cellular level, glial fibrillary acidic protein-positive (GFAP+) astrocytes were significantly increased in the lesioned dorsolateral striatum of PD rats compared with the intact side and the sham group. Prolonged L-DOPA treatment further increased GFAP levels. Neither 6-OHDA damage nor L-DOPA treatment influenced the striatal expression of vascular endothelial growth factor (VEGF). Additionally, there was a considerable increase in synapse-associated proteins (SYP, PSD95, and SAP97) in the lesioned striatum of LID rats relative to the PD rats. Golgi-Cox staining analysis of the dendritic spine morphology revealed an increased density of dendritic spines after chronic L-DOPA treatment. Taken together, our findings suggest that striatal volume changes in LID rats involve astrocyte activation, enrichment of synaptic ultrastructure and signaling proteins in the ipsilateral striatum. Meanwhile, the data highlight the enormous potential of structural MRI, especially VBM analysis, in determining the morphological phenotype of rodent models of LID.

## Introduction

Levodopa (L-DOPA)-induced dyskinesia (LID) is a common motor complication of chronic L-DOPA treatment in patients with Parkinson’s disease (PD) (Iravani and Jenner, 2011). It significantly reduces the therapeutic efficacy and adversely affects the patient’s quality of life. Research efforts to uncover the neuroanatomical correlates of LID in the clinical and in relevant animal models of LID are vital to help elucidate the pathogenesis of LID and facilitate LID treatment (Brotchie et al., 2005). Several studies using functional magnetic resonance imaging (fMRI) (Herz et al., 2016) and positron emission tomography (PET) (Rascol et al., 1998; Brooks et al., 2000) have reported that LID involves molecular changes, metabolism, and abnormal brain network connections in the cortex-striatum-cortex loop. Electrophysiological and neuropathological studies have suggested that LID results from long-term adaptive brain plasticity (Belujon et al., 2010; Ohlin et al., 2011; Zheng et al., 2020), which could be attributed to neuroanatomical remodeling at the level of cells (Bortolanza et al., 2015; Mulas et al., 2016; Fletcher et al., 2020), spine and synapses (Zhang et al., 2013; Suarez et al., 2016; Fieblinger et al., 2018), or blood vessels (Lindgren et al., 2009; Ohlin et al., 2011; Booth et al., 2021). It remains unclear whether these cellular contributors cause macroscopic structural changes in the brain volume of LID. This is relevant, since neuroimaging studies both in human (Zatorre et al., 2012; Fauvel et al., 2014) and rodent (Biedermann et al., 2012; Dodero et al., 2013) have demonstrated a linear relationship between functional activity and brain structure. Structural MRI studies have shown significantly progressive cortical and subcortical atrophy in patients with PD than in healthy controls (Filippi et al., 2020; He et al., 2020). Additionally, compared with non-dyskinetic patients, dyskinetic patients with PD present with macroscopic structural changes in brain volume, including increased thickness of the right inferior frontal sulcus and increased gray matter volume (GMV) and thickness of the inferior frontal cortex (Cerasa et al., 2011, 2013a,2013b). However, there have been no positive results regarding the striatum, which is crucially involved in the pathogenesis of LID (Belujon et al., 2010; Iravani et al., 2012). Moreover, the precise cellular mechanisms underlying the neuroanatomical changes remains unclear due to the limited resolution in human imaging studies. Combining well-validated animal models of LID with advanced non-invasive and accessible structural MRI methods (Duty and Jenner, 2011; Fletcher et al., 2020) could overcome this limitation and allow direct integration of different research areas, accelerate the clinical translation of basic findings, and facilitate explanation of MRI phenomena in dyskinetic patients (Finlay et al., 2014).

This study aimed to map neuroanatomical changes in a rodent model of LID based on voxel-based morphometry (VBM) analysis, which is an automated whole-brain morphometry technique (Ashburner and Friston, 2000) and has been widely used in many central nervous system diseases (Madeira et al., 2020; Riederer et al., 2020; Takano et al., 2020). Compared with the traditional manually delineated, anatomically defined regions of interest (ROI) (Kubicki, 2002) and the surface based morphometric analysis that only detects cortical thickness and sulcus depth (Tso and Goadsby, 2015), VBM can provide a wider range of brain information, and much faster and less time-consuming.

We hypothesized that chronic exposure to L-DOPA could alter the MRI phenotype. If true, we sought to assess the possible mechanism underlying these changes.

## Materials and Methods

### Animals

Adult male Sprague–Dawley rats (weight: 180–200 g) were purchased from Beijing Sibeifu Biotechnology Co., Ltd., China. All rats were housed in a specific-pathogen-free environment under controlled conditions (22 ± 1°C, 55–65% humidity, 12-h light/dark cycle) and adequate food and water, they were allowed to acclimate for 1 week before study initiation. The experiment was approved by the Institutional Animal Care and Use Committee at Tongji Medical College, Huazhong University of Science and Technology, China.

### Chemicals

6-hydroxydopamine (6-OHDA), ascorbic acid, apomorphine hydrochloride, L-DOPA methyl ester, and benserazide were purchased from Sigma-Aldrich. 6-OHDA (2 μg/μL) and apomorphine (0.1 mg/mL) were dissolved in sterile saline containing 0.02% ascorbic acid. L-DOPA (12 mg/mL) and benserazide (6 mg/mL) were directly dissolved in sterile saline before use.

### 6-Hydroxydopamine Lesions and Apomorphine-Induced Rotations

The rats were deeply anesthetized with isoflurane (3% induction and 1.5–2% maintenance in pure oxygen) and fixed on a stereotaxic apparatus. A temperature controller system was used to keep the rats warm during the operation. For 6-OHDA Lesioned rats (*n* = 40), a total dose of 8 μg of 6-OHDA (2 μg/μL, 4 μL, Sigma) was injected into two points (with minor modifications) of the right medial forebrain bundle through a 10-μL microsyringe at 1 μL/min. The stereotactic coordinates were as follows: anteroposterior (AP), −4.4 mm; mediolateral (ML), −1.5 mm; and dorsoventral (DV), 7.8 mm from dura and AP, −4.4 mm; ML, −1.5 mm; DV, 7.9 mm from dura (Chen et al., 2017). The sham group (*n* = 12) was injected with equal saline amounts containing 0.02% ascorbic acid at the same location. 400,000 units/kg/d of penicillin (3 days) was intramuscularly injected to prevent infection post-surgery. One rat died after surgery. After 2 weeks of recovery, apomorphine (0.05 mg/kg s.c.)-induced contralateral rotations were recorded to assess the efficacy of the dopaminergic lesion. Only rats with >200 turns contralateral to the lesioned side within 30 min were considered eligible PD models with nearly complete lesions (Schwarting and Huston, 1996; Paille et al., 2010) and were chosen for subsequent study.

### Treatments

**FIGURE 1:**
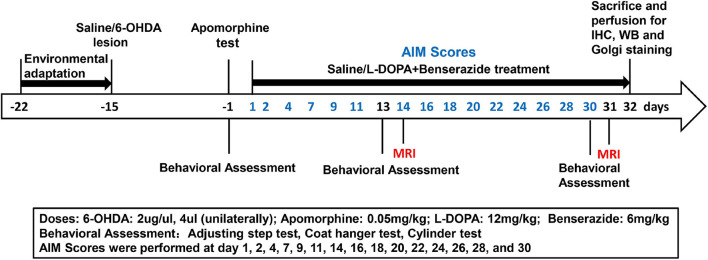
Timeline for induction and evaluation of L-DOPA-induced dyskinesia in rats with 6-OHDA lesions. Behavioral assessment was performed at 2 weeks post surgery and at 2-weeks intervals after L-DOPA/saline treatment. MRI was performed at 2 weeks and 1 month. AIMs scores were assessed according to the time noted in the figure. All animals were sacrificed after the last MRI test for subsequent analysis.

Two days after the apomorphine test (Figure 1, Supplementary Figure 1), three eligible PD rats were used for tyrosine hydroxylase (TH) staining to evaluate dopaminergic depletion in the striatum and substantia nigra (SN) after 6-OHDA lesions. The remaining 24 successful hemiparkinsonian rats were randomly divided into the PD + saline group (*n* = 11) and PD + L-DOPA group (*n* = 13). The rats were injected daily with either L-DOPA [12 mg/kg with benserazide (6 mg/kg), i.p.] or saline (12 mg/kg, i.p.). After the chronic dyskinesia induction phase (21 days), L-DOPA was administered 2–4 times per week to maintain stable reproducible abnormal involuntary movements (AIMs) (Iderberg et al., 2015). The sham group (*n* = 12) received a similar volume of saline injections. During the priming period, L-DOPA-induced AIMs were regularly recorded (as shown in Figure 1) by a blinded examiner as an index of dyskinesia.

### Behavioral Assessment

#### Adjusting Step Test

The stepping test was performed 2 weeks post-surgery and 15 min before (pre-) and after (post-) saline/L-DOPA treatment on days 13 and 30, as previously described (Chang et al., 1999). The rat’s hindquarters and contralateral forelimb were slightly lifted from the table by the experimenter, with only the test forelimb touching the table. The number of adjusting steps of both forelimbs in the forward direction over a distance of 90 cm within 5 s was counted (Pinna et al., 2007; Chen et al., 2017). The trial was conducted thrice with the average value being recorded.

#### Coat Hanger Experiment

The coat hanger experiment was conducted as described previously, with slight modifications (Moran et al., 1995; Voikar et al., 2002). The rats were naturally placed at the center of the coat hanger (diameter, 3 mm; horizontal length, 35 cm; 40 cm away from the ground) on their forepaws. The body position of each rat was observed for 30 s, with the trial being repeated thrice. The scores were assigned as follows: 0, falling off within 10 s; 1, two forepaws remained on the hanger; 2, similar to 1, but with attempts to climb the hanger; 3, both forepaws plus at least one hind paw on the hanger; 4, all four paws plus tail wrapped around the hanger; and 5, escape to the edges of the hanger balance shaft. Moreover, we measured the latency of the rats to fall off. The mean value of the three experiments was calculated for statistical analysis.

#### Cylinder Test

A modified version of the cylinder test was used to measure asymmetry forelimb use in the spontaneous exploration of the walls of a cylindrical enclosure, which is a common motor function test after unilateral 6-OHDA injury (Schallert, 2006). The test was performed at 2 weeks post-surgery and 15 min before (pre-) and after (post-) saline/L-DOPA treatment on days 13 and 30. In the cylinder test, the animals were individually placed in a clear plexiglass cylinder in a dimly lit room (21 cm, diameter; 16 cm, height) and observed for 5–10 min depending on the activity of the rats. The time in the cylinder was either limited to 20 supporting front paw contacts or until the time reached 10 min. This relatively short interval (15 min) was chosen to ensure that the forelimb usage preferences could be tested in all rats, regardless of the dyskinesia severity. The number of supporting wall contacts during vertical exploration in the lesioned (contralateral), unlesioned (ipsilateral), or bilateral was recorded in a given trial. Data were expressed as the percentage of lesioned forelimb use: [(lesioned + 1/2 bilateral) divided by (lesioned + unlesioned + bilateral)] × 100, as previously described (Schallert, 2006). Further, we demonstrated unilateral and bilateral wall contact.

### Abnormal Involuntary Movements Score

For quantification of AIMs, the rats were individually checked for 1 min at 35-min intervals for a total of 140 min after L-DOPA or saline treatment on days 1, 2, 4, 7, 9, 11, 14, 16, 18, 20, 22, 24, 26, 28, and 30 using the validated AIMs scale. As previously described (Winkler et al., 2002; Chen et al., 2017), the AIM subtypes (axial, limb, and orofacial dyskinesia, also named ALO dyskinesia) were divided according to the severity into grades 0–4 as follows: 0, absent; 1, occasional, present <50% of the time; 2, frequent, present in >50% of the time; 3, continuous throughout but interrupted by external stimuli; and 4, continuous, not interrupted by external stimuli (Lundblad et al., 2004; Chen et al., 2017). The maximum total ALO dyskinesia score was 48. Rats that consistently displayed a severity score of 3–4 in no less than two AIM subtypes in at least two monitoring testing sessions were classified as dyskinetic (LID) and selected for further analysis (Westin et al., 2006).

### Magnetic Resonance Imaging

Two weeks and 1 month after the L-DOPA/saline treatment (Figure 1), T2-weighted MRI experiments were performed on a 7.0T small-animal magnet MRI scanner (Bruker BioSpin, Ettlingen, Germany). A 72-mm-diameter volume coil and quadrature surface coil were used for radiofrequency pulse transmission and signal detection, respectively. T2-weighted anatomical images were obtained with 39 contiguous coronal slices using Rapid Acquisition with Relaxation Enhancement (RARE) sequence based on the following parameters: repetition time, 4,000 ms; effective echo time, 36 ms; matrix size, 128 × 128; field of view, 30 mm × 30 mm; slice thickness, 0.5 mm; and RARE factor 4 and 8 averages. During the MRI experiments, the rats (*n* = 10 per group) were anesthetized with isoflurane (3% induction and 1.5–2% maintenance in pure oxygen) and placed on the animal bed with a tooth bar and ear bars for head immobilization. The body temperature was maintained at 37°C by a warm water circulator system to avoid the influence of gas anesthesia. The respiration rate was continuously monitored throughout the experiment.

Voxel-based morphometry analysis was chosen to assess GMV differences between groups with data processing pipelines provided by Statistical Parametric Mapping 12 (SPM12)^1^. Using diffeomorphic anatomical registration through exponentiated lie algebra, T2-weighted anatomical images were segmented and spatially normalized into a reference space defined by a set of custom-built tissue probability templates with a spatial resolution of 125 μm × 125 μm × 125 μm. Modulated GM maps were obtained from each animal. These maps were smoothed using a 0.7-mm full width at half maximum Gaussian kernel and compared voxel-wise with two-sample *t*-tests. The level of significance was set at *p* < 0.001, uncorrected, cluster size = 50.

### Tissue Preparation

To minimize the number of animals used, after the MRI experiments, the same batch of rats were decapitated under anesthesia with isoflurane for subsequent experiments. The striata of four rats from each group were rapidly removed on ice and immediately preserved at −80°C for biochemical evaluations; further, another three freshly lesioned striata from each group were extracted for Golgi staining. Moreover, three rats of each group were transcranially perfused with 0.9% saline followed by 4% ice-cold paraformaldehyde; then the whole brains were completely removed and fixed in 4% paraformaldehyde at 4°C for 48 h. The fixed samples were dehydrated, embedded, and sectioned for immunohistochemistry (IHC).

### Western Blotting

Striatal tissues were dissected and homogenized for total protein extraction. Lysates were centrifuged at 12,000 × *g* at 4°C for 15 min, followed by measurement of protein concentrations using the bicinchoninic acid assay kit (Biosharp, China). Equal protein amounts (40 μg) from each sample were separated on a 10 or 12% SDS-PAGE gel. Subsequently, they were transferred to a polyvinylidene difluoride membrane (Millipore, United States), followed by blocking in 5% non-fat milk or 5% bovine serum albumin (BSA) for 1.5 h at room temperature. After washing, the membranes were incubated using the following primary antibodies overnight at 4°C: anti-TH Rabbit pAb (Tyrosine hydroxylase, 1:3000, Proteintech, 25859-1-AP); anti-GFAP Mouse mAb (glial fibrillary acidic protein, 1:2500, Servicebio, GB12096); anti-VEGF Mouse mAb (vascular endothelial growth factor, 1:200, Santa Cruz, sc-7269); anti-SYP Rabbit pAb (synaptophysin, 1:10000, Servicebio, GB11553); anti-PSD95 Rabbit pAb (post synaptic density protein 95, 1:500, Servicebio, GB11277); anti-SAP97 Rabbit pAb (synapse-associated protein 97, 1:2000, Abcam, ab3437); anti-GAPDH Rabbit pAb (glyceraldehyde 3-phosphate dehydrogenase, 1:10000, GeneTex, GTX100118). The day after incubation, the membranes were washed thrice, then incubated with the appropriate secondary horseradish peroxidase (HRP)-conjugated antibodies for 1 h at room temperature: goat anti-rabbit IgG (1:10000, Abbkine, A21020) or goat anti-mouse IgG (1:10000, Abbkine, A21010). After washing, we used enhanced chemiluminescence kits (Biosharp, BL520A) to dye the membranes; moreover, bands were detected using a fluorescent chemiluminescence gel imaging system (Syngene, United Kingdom). ImageJ software was used to analyze band intensities.

### Immunohistochemistry

Immunohistochemistry was performed as described previously (Tan et al., 2020). Briefly, paraffin-embedded brains were cut at a 4-μm thickness in the coronal plane and mounted on slides. After deparaffinization and rehydration, the sections were baked in the basic antigen retrieval buffer (pH 9.0). After rinsing thrice using phosphate-buffered saline (pH 7.4), the sections were blocked with 5% BSA (Sigma, United States) for 30 min at room temperature, then incubated with the following primary antibodies overnight at 4°C: rabbit anti-TH (1:200), mouse anti-GFAP (1:700), mouse anti-VEGF (1:50), rabbit anti-SYP (1:1000), and rabbit anti-PSD95 (1:50). Subsequently, the sections were washed and incubated with HRP-labeled secondary antibodies for 1 h at room temperature, followed by visualization with 3,3-diaminobenzidine solution, counterstaining using Harris hematoxylin, dehydration, and coverslipping. An Olympus camera connected to a microscope was used to obtain images under the same light intensity, followed by analysis using ImageJ software.

### Golgi-Cox Impregnation and Dendritic Spine Analysis

To further explore whether striatal morphological changes on MRI in LID rats are associated with changes in dendritic spine, we performed morphological studies using the FD Rapid GolgiStain^TM^ Kit (FD NeuroTechnologies, Columbia, MD, United States) following the manufacturer’s instructions, with slight modifications (Du, 2019). Briefly, the animals were deeply anesthetized, then the brains were integrally removed to avoid tissue damage without perfusion. After rinsing with distilled water, the brains were hemisected using a blade, and the lesioned striata were sliced into approximately 8-mm thick blocks. First, the blocks were immersed in impregnation solution (A and B), which was replaced the next day; subsequently, they were kept in the dark for 4 weeks at room temperature. Next, tissues were transferred into solution C, after 24 h, the fluid was replaced and stored in the dark for the next week. A vibratome was used to cut a series of 100-μm thick slices. Subsequently, the sections were mounted onto gelatin-coated microscope slides. After naturally drying, the slices were stained, dehydrated, and cover-slipped with permount. The dendritic spines were analyzed on the dorsolateral striatum between bregma 2.0 and 0.5, with obvious morphological changes on MRI. Secondary dendrites with a length of ≥10 μm without obvious shielding were selected for photography. There were 5–6 neurons included, and a total of 30 dendritic fragments were used to analyze dendritic spines in each animal. Optical images were obtained using a camera connected to a Nikon Eclipse ci microscope (Nikon, Tokyo, Japan) at the same light intensity by an independent group-blinded experimenter. Results were expressed as the number of dendritic spines per 10 μm in length.

### Statistical Analysis

All data were analyzed using GraphPad Prism 8.0 software. The paired-samples *t*-test was used to compare behavioral changes before and after treatment within the same group; further, between-group comparisons were performed using an independent-sample Student’s *t*-test. One-way analysis of variance (ANOVA) was conducted for comparisons between the three groups. Among-group differences in the percentage of forelimb use before and after treatment at day 13 and day 30, and intra-group behavior differences between day 13 and day 30 were analyzed using two-way ANOVA, followed by Bonferroni’s *post hoc* test. *P* < 0.05 was considered statistically significant. All data are presented as mean ± SEM or boxplot showing the median, quartiles, and ranges.

## Results

### Validation of the Unilateral 6-Hydroxydopamine-Lesioned Rat Model

**FIGURE 2:**
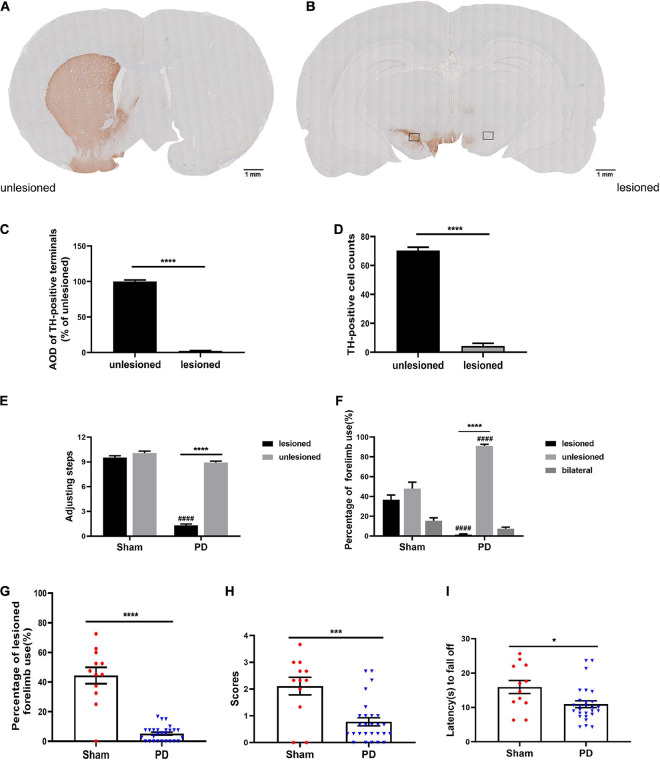
Evaluation of a PD rat model with complete unilateral damage at 2 weeks post surgery. **(A,B)** Tyrosine hydroxylase (TH) immunostaining in the striatum and SN of the 6-OHDA lesioned rats. **(C,D)** Analysis of TH differences (AOD, average optical density) between the unlesioned and lesioned side (scale bars: 1 mm). The boxes represent the SN region (area 0.2 mm^2^) where positive neurons are counted. *N* = 3/group. **(E–I)** Behavioral assessment of each group. **(E)** Adjusting steps test. **(F,G)** Cylinder test. **(H,I)** Coat hanger experiment. *p < 0.05, ***p < 0.001, ****p < 0.0001 vs. the specified group.^ ####^*p* < 0.0001 vs. the ipsilateral side in the sham group. *N* = 12 (sham); *N* = 27 (PD). Error bars represent SEM.

After acute challenging with apomorphine (0.05 mg/kg, s.c.), 27 out of 40 (67.5%) 6-OHDA-injected rats presented with >200 contralateral rotations, which was considered as the completely damaged PD model. Contrastingly, the sham rats did not present with rotating behavior. Additionally, TH immunostaining revealed almost complete depletion (TH-positive striatal dopaminergic terminals, Figures 2A,C; dopaminergic neurons in the SN, Figures 2B,D; *n* = 3) in the lesioned side, which was consistent with the hemiparkinsonian model. Subsequent behavioral test results (Figures 2E–I) confirmed severe sensorimotor deficits in the PD group. The typical performances were significant decrease in the number of steps on the contralateral limb of the lesion (Figure 2E), lower preference in using the contralateral anterior limb (Figures 2F,G), and impaired coordination ability (Figures 2H,I) compared with those in the sham group.

### Chronic Levodopa Administration Induced Abnormal Involuntary Movements While Improved Movement Deficits in the 6-Hydroxydopamine-Lesioned Rats

**FIGURE 3:**
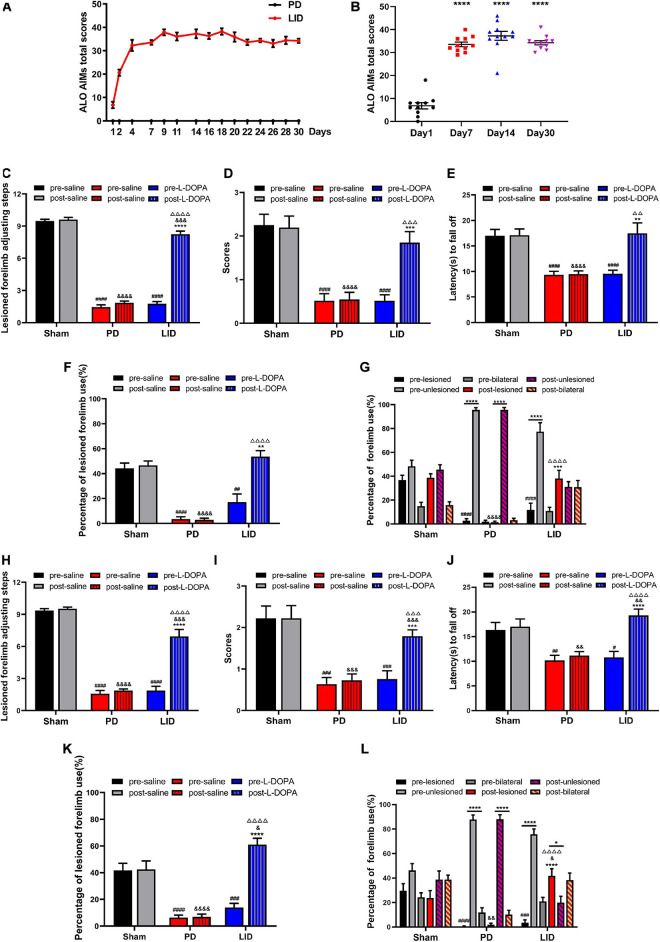
L-DOPA treatment can improve motor symptoms and induce dyskinesia (LID). **(A,B)** Behavioral characteristics of the LID rat model. **(A)** LID was induced through multiple injections of levodopa and benserazide into PD rats. The figure shows the total of the axial, limb, and orofacial scores (ALO AIMs) obtained at 35-min intervals within 140 min after administration of L-DOPA or saline. The maximum theoretical single-time highest score is 48. The AIMs score gradually increased from day 1 to day 7 and plateaued after day 7. The saline-treated PD rats lacked abnormal behavior. **(B)** Changes in the total score of ALO AIMs at different time points in the LID group. ****P < 0.0001 vs. Day 1. *N* = 11/group. **(C–L)** Behavioral assessment [Adjusting step test **(C,H)**, Coat hanger experiment **(D,E,I,J)** and Cylinder test **(F,G,K,L)**] of each group at day 13 **(C–G)** and day 30 **(H–L)**. L-DOPA administration ameliorated behavioral deficits versus saline-treated rats. *p < 0.05, **p < 0.01, ***p < 0.001, ****p < 0.0001 vs. pre-L-DOPA/pre-lesioned LID group or the specified group;^ #^*p* < 0.05, ^##^*p* < 0.01, ^###^*p* < 0.001, ^####^*p* < 0.0001 vs. the pre-saline/pre-lesioned sham group; ^&^*p* < 0.05, ^&&^*p* < 0.01, ^&&&^*p* < 0.001, ^&&&&^*p* < 0.0001 vs. the post-saline/post-lesioned sham group; ^ΔΔ^ *p* < 0.01, ^ΔΔΔ^ *p* < 0.001, ^ΔΔΔΔ^ *p* < 0.0001 vs. the post-saline/post-lesioned PD group. Error bars represent SEM. *N* = 12 (sham); *N* = 11 (PD); *N* = 11 (LID).

After the behavioral tests, remaining 24 rats with full lesions induced by 6-OHDA were randomly allocated to the saline (*n* = 11) or L-DOPA (*n* = 13) treated groups. As shown in Figures 3A,B, chronic administration of L-DOPA plus benserazide, 85% (11/13) to 6-OHDA lesioned rats yielded severe and reproducible dyskinetic ALO AIMs (axial, orofacial, and limb AIMs) and were considered as the LID model. The AIMs score increased gradually from day 1 to day 7, and plateaued after day 7. Two rats were eventually excluded because they exhibited mild and occasional AIMs. None of the saline-treated 6-OHDA lesioned rats showed AIMs. To observe the treatment effect over time, behavioral assessments were conducted 15 min before (pre-) and after (post-) L-DOPA/saline treatment at day 13 and day 30, respectively. As shown in Figures 3C–L, L-DOPA administration significantly improved motor dysfunction in the LID group; contrastingly, the other two groups performed similarly before and after treatment at both two time points. During the test, we failed to observe significant behavioral differences (including Adjusting step test, Cylinder test and Coat hanger experiment) between day 13 and day 30 in each group, both before and after the intervention (Supplementary Figure 2).

### Chronic Levodopa Treatment Produced a Progressive Pattern of Brain Structural Changes

**FIGURE 4:**
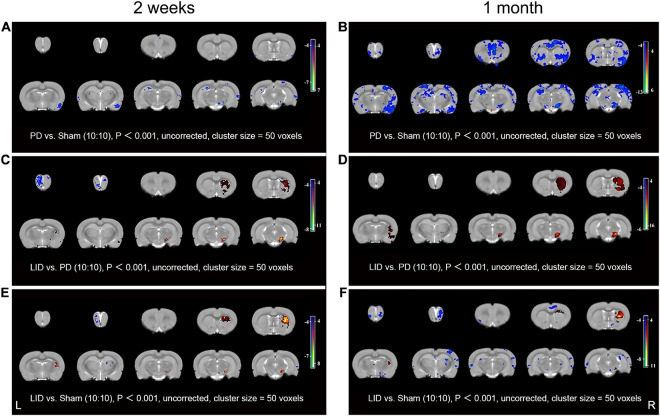
VBM analysis results at 2 weeks **(A,C,E)** and 1 month **(B,D,F)** after L-DOPA/saline administration. **(A)** Compared with the sham group, the PD group showed significantly decreased volumes of the SN in the lesioned side, as well as parts of the bilateral cortices and hippocampus, at 2 weeks. **(B)** These changes were more obvious and had a wider range at 1 month. Specifically, reduced volume in the bilateral striatum was newly observed. Notably, the whole-brain analysis did not reveal increased regional volume. The LID group showed increased volume mainly in the dorsolateral striatum, piriform cortex, and SN of the lesioned side relative to the PD group at 2 weeks **(C)**; moreover, there was a more prominent increase at 1 month **(D)**, additionally, the volume decrease in the bilateral olfactory bulbs at 2 weeks disappeared at 1 month. **(E,F)** These volume changes were partly reversed by L-DOPA treatment. Decreased and increased regional volumes are displayed in blue and red, respectively. L, unlesioned side; R, lesioned side (*N* = 10/group, *P* < 0.001).

We used structural MRI to determine whether chronic exposure to L-DOPA contributed to macro-structural changes in rat brain. Given the limitation of MRI acquisition time, only 10 rats in each group were included in this experiment. Structural MRI was performed at 2 weeks and 1 month after treatment. VBM analysis was used to detect and analyze dynamic changes in brain volume. Compared with the sham groups, there was a significant GMV decrease in the piriform cortex, SN, and visual cortex in the ipsilateral (lesioned) hemisphere in the PD group at 2 weeks. Additionally, there were small clusters of significant GMV decrease in the bilateral ectorhinal cortices, thalamus, and hippocampus. No brain region showed increased GMV (Figure 4A). The scope of the lesions gradually increased and progressively became more obvious. Specifically, there was a reduced volume in the bilateral striatum at 1 month (Figure 4B). Contrastingly, visual analysis of MR images revealed a progressive GMV increase in the dorsolateral striatum, piriform cortex, and SN of the lesioned hemisphere, which was accompanied by significantly decreased GMV in the bilateral olfactory bulbs at 2 weeks after treatment in the LID group than in the PD group (Figure 4C). These changes were more prominent at 1 month, while the decrease in the bilateral olfactory bulbs disappeared (Figure 4D). Compared with the sham group, the LID group showed a volume decrease in a few scattered brain regions (such as the olfactory bulbs and ectorhinal cortices), as well as a significant increase in the volume of the damaged dorsolateral striatum (Figures 4E,F), which could be attributed to the neutralization effect. More brain volume information of relevant brain regions can refer to in the Supplementary Table 1.

### Effects of Levodopa Administration on Astrocytes, Vasculogenesis, and Synaptic Plasticity

**FIGURE 5:**
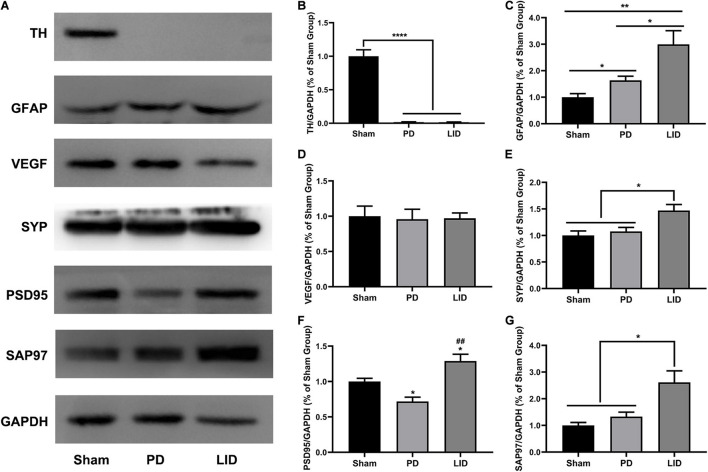
L-DOPA treatment significantly increased the levels of GFAP and synaptic-associated proteins (SYP, PSD95, SAP97), but not vasculature, in the lesioned striatum. **(A)** Representative western blots of TH, GFAP, VEGF, and synaptic associated proteins in the lesioned striatum of the three groups. GAPDH served as the internal control. **(B–G)** Quantitative analysis of the aforementioned proteins. Data were presented as% of the sham group. *P < 0.05, **P < 0.01, ****P < 0.0001 vs. the specified or sham group; ^##^*P* < 0.01 vs. the PD group. All results were presented as mean ± SEM (*N* = 4/group).

**FIGURE 6:**
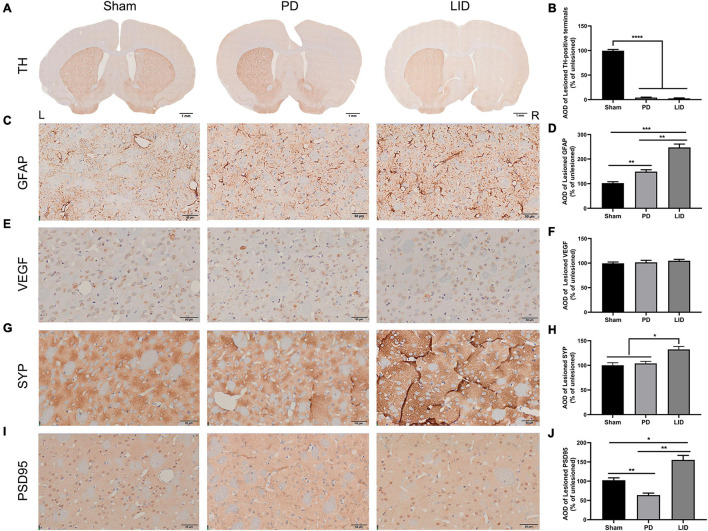
L-DOPA treatment promoted astrocytic and synaptic pathology in the lesioned striatum. **(A)** TH immunostaining of the striatum in each group. **(C,E,G,I)** Representative immunohistochemical images of GFAP, VEGF, and synaptic associated proteins (SYP, PSD95) of the dorsolateral striatum from the three groups in the lesioned side. [Scale bars: **(A)** is 1 mm, **(C,E,G,I)** is 50 μm]. **(B,D,F,H,J)** Analysis of differences of the aforementioned proteins. AOD, average optical density. Results were expressed as % of the unlesioned side. *P < 0.05, **P < 0.01, ***P < 0.001, ****P < 0.0001 vs. the specified group. Error bars represent SEM (*N* = 3/group).

We conducted post-mortem investigations to assess underlying cellular mechanisms of the VBM changes in MRI after different interventions. Neuronal and extraneuronal changes, including neurons, glia, microvasculature, synaptic density, and extracellular space, may affect MRI signal measurement, which is sensitive to changes in water proton properties (Perea et al., 2009; Herz et al., 2014). Since the striatum is crucially involved in the pathogenesis of LID (Iravani et al., 2012) and showed the most obvious volume changes, we mainly focused on striatal pathological analysis. Western blotting and IHC of TH in the PD and LID rats demonstrated marked striatal dopaminergic denervation (Figures 5A,B, 6A,B), which further confirmed the success of the model.

### Levodopa Treatment Further Increased the Levels of Activated Astrocytes

Astrocytes, which are involved in the inflammatory response of the central nervous system, are considered to be involved in LID occurrence (Bortolanza et al., 2015; Pisanu et al., 2018). Accordingly, we estimated striatal glial activation. As shown in Figures 5A,C, western blotting of the lesioned striatum revealed that activated astrocytes were involved in PD and LID development. Consistent with these findings, IHC revealed a significant increase in GFAP+ astrocytes in the lesioned dorsolateral striatum than in the intact side in both PD and LID groups (data not shown). Compared with the sham group, the PD and LID groups showed significantly increased density of GFAP+ cells in the lesioned side, which was further increased by chronic L-DOPA administration (Figures 6C,D).

### Levodopa Treatment Did Not Alter the Microvasculature of the Rat Striatum Injured by 6-Hydroxydopamine

Rodent and human studies have reported angiogenesis in the denervated dyskinetic striatum (Ohlin et al., 2011; Muñoz et al., 2014; Jourdain et al., 2017), which could contribute to the increased volume. Accordingly, we investigated the levels of VEGF, which is crucially involved in angiogenesis and the sprouting of new capillaries during development (Carmeliet, 2000). However, we observed no significant differences in the level of VEGF between the LID group and PD group. Similarly, 6-OHDA lesions did not affect vasculature (Figures 5A,D), which was confirmed through pathological analysis (Figures 6E,F).

### Levodopa Treatment Induced Aberrant Synaptic Plasticity in 6-Hydroxydopamine Parkinsonian Rodents

**FIGURE 7:**
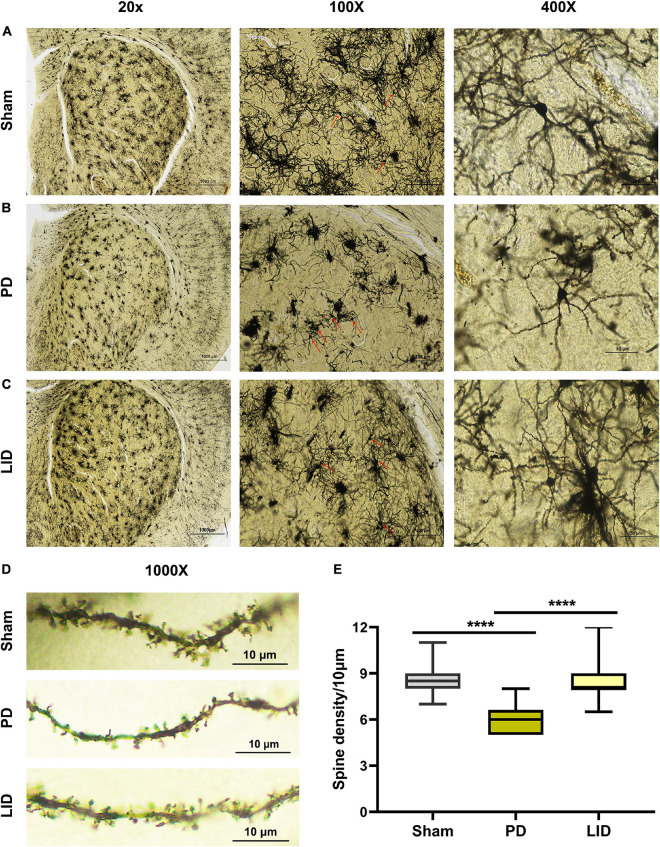
Chronic L-DOPA treatment reversed the reduced dendritic spine density in the lesioned striatum in PD rats. **(A–D)** Representative microphotographs of lesioned striatum stained using the FD Rapid GolgiStain^TM^ kit. At low magnification (20×), the integral field-vision of the striatum is primarily composed of regularly distributed cluster cells, whose staining mainly corresponded to glial cells. Medium spiny neurons (MSN, arrow; 100×) scattered in the middle of numerous tufted glial cells. A higher magnification (400×) shows a typical MSN with spinous dendrites protruding in all directions. The morphology and density of dendritic spines are visible (1000×). **(E)** As shown, the dendritic spine density in the PD group was much lower, compared with the sham group; moreover, the LID group showed a significantly higher dendritic spine density than in the PD group. ****P < 0.0001 vs. the specified group. The data were presented as boxplot showing median, quartiles and ranges (*N* = 3/group).

Human and rodent studies have demonstrated aberrant synaptic plasticity in the cortical basal ganglia motor circuits in PD (Tang et al., 2001; Prescott et al., 2009; Kawashima et al., 2013; Thiele et al., 2014; Tozzi et al., 2021). Therefore, we investigated whether chronic L-DOPA treatment could change synaptic plasticity through western blotting and IHC for detecting the levels of several synapse-related proteins. As shown in Figures 5A,E–G, 6G–J, L-DOPA treatment increased synaptic proteins (SYP, PSD95, and SAP97) in the lesioned striatum of LID rats than in saline-treated PD rats. There were no significant differences in the SYP and SAP97 levels between the PD and sham groups. However, PSD95 levels were much lower in PD rats than in sham rats. Given that aberrant synaptic plasticity in striatal medium spiny neurons (MSNs) and cortical pyramidal neurons is often accompanied by morphological changes in LID models (Zhang et al., 2013; Suarez et al., 2016), we performed morphological analysis to determine the cause of striatal volume changes detected by VBM. The microstructure of the dendritic spines was observed through Golgi staining (Du, 2019). As expected, 6-OHDA lesions significantly decreased dendritic spines in the PD group relative to the sham group, which was dramatically increased by chronic L-DOPA treatment (Figure 7). Taken together, these findings demonstrated that L-DOPA administration promoted maladaptive synaptic plasticity.

## Discussion

The pathogenesis of LID is not fully understood. Although many attempts in functional neuroimaging and some results have been achieved (Cerasa et al., 2015; Herz et al., 2015), dyskinesia interferes with signal acquisition in experimental animals and limits the utility of fMRI, which provides us an opportunity to address this issue from an anatomical perspective. The striatum is an important hub of the cortical-basal ganglia circuit (Picconi et al., 2018b). It is believed that both the functional and neuroanatomical change of the striatum influence the signal transmission of the whole-circuit, and contribute to the onset of LID. Moreover, striatal volume changes are most obvious on MRI after long-term L-DOPA treatment in our study. Therefore, we mainly focused on the pathological analysis of the striatum here.

The main findings of this study are that VBM analysis observed a specific pattern of significant GMV decreases mainly in several cortical and subcortical regions of the lesioned hemisphere, including the site of the primary lesion (substantia nigra) following 6-OHDA lesioning (Figure 4A). This pattern of GMV decreases extended to more extensive areas of both hemispheres (including bilateral striatum) over time (Figure 4B). No significant GMV increases were noted. Meanwhile, remarkable movement deficit was observed (Figures 2E–I, 3C–L). Chronic L-DOPA treatment partially reversed these changes, with volume reduction observed only in a few scattered brain regions. The volume of the dorsolateral striatum on the lesioned side was still significantly increased (Figures 4E,F). In addition, long-term L-DOPA treatment induced abnormal involuntary movements (Figures 3A,B) and resulted in significant increases in the volume of the ipsilateral striatum, piriform cortex, and SN compared with those in saline-treated PD rats (Figures 4C,D). Regarding striatal cellular level, there was a prominent denervation of the dopaminergic (TH-positive) fibers (Figures 5A,B, 6A,B), as well as a decrease in PSD95 (Figures 5A,F, 6I,J) and dendritic spines (Figure 7) in 6-OHDA lesioned rats. Moreover, chronic L-DOPA administration increased GFAP + astrocytes of the lesioned striatum, compared with saline-treated PD rats (Figures 5A,C, 6C,D). In addition, there were lesion-associated increases in synapse-associated proteins (Figures 5E–G, 6G–J). Consistent with this, there was a remarkable increase in the dendritic spine density in LID rats. Notably, neither 6-OHDA injury nor L-DOPA treatment affected striatal VEGF expression (Figures 5A,D, 6E,F). Overall, our findings confirmed that chronic L-DOPA administration is associated with striatal structural changes, which could involve astrogliosis and altered synaptic plasticity. Conceivably, these findings may shed light into the pathology of LID, allowing us one step closer to dissect the molecular mechanism of how chronic L-DOPA triggers LID.

Our preliminary MRI findings revealed that L-DOPA administration caused macroscale changes in the GMV in rodents, which is consistent with previous structural MRI findings in dyskinetic patients with PD (Cerasa et al., 2011, 2013a,2013b). Notably, although previous studies have not reported striatal volume changes, there have been reports of region-specific increases in GMV and cortical thickness, especially in the prefrontal cortex, after L-DOPA treatment in dyskinetic patients with PD than in non-dyskinetic patients. In this study, compared with PD rats, LID rats showed a significant volume increase in the lesioned dorsolateral striatum. This is consistent with the findings of a recent structural MRI study *in vitro* (Fletcher et al., 2020), which reported an absolute volume expansion in the lesioned striatum of chronic L-DOPA-treated rats, compared with saline-treated rats. Moreover, this previous study reported a positive correlation between volume changes and AIMs scores, which suggested that microglia may be involved in this process. However, it remains unclear whether MRI can detect morphological changes in other brain regions after repeated L-DOPA treatment and whether there are dynamic changes over time. The present dynamic MRI study *in vivo* provides important evidence for the progressive pattern of whole brain structural changes in LID rats and demonstrates the potential relationship between macroscopic variations and microscopic cellular parameters.

Several studies on different neurotoxin PD models have demonstrated bilateral GMV reduction on a topographic scale, including in the striatum and SN (Vernon et al., 2010; Westphal et al., 2016; Modo et al., 2017). In our study, MRI revealed a similar phenotype in the 6-OHDA rat model, with these changes worsening over time. This suggests that cortical and subcortical GMV reduction is a distinctive characteristic of nigrostriatal degeneration. These changes may explain the motor deficits in 6-OHDA rats. We could not directly explore the relationship between MRI-derived GM atrophy and parkinsonian sensorimotor deficits, which was beyond our study scope. Notably, a previous study reported overactivation of the bilateral sensorimotor cortex upon stimulation of the left or right forepaw (Pelled et al., 2002). Another study reported that intracortical microstimulation bilaterally reduced M1 excitability. However, this reduction was greater in the damaged hemisphere than in the intact hemisphere (Viaro et al., 2011). Taken together, the between-hemisphere interaction is crucially involved in the pathophysiology of unilateral 6-OHDA rats. Further, our findings suggest that such volume changes could be at least partly reversed by L-DOPA treatment.

Literature has reported that hippocampal volume increase in mice after voluntary exercise could be attributed to astrogliosis (Biedermann et al., 2016). Considering the astrocytes may be crucially involved in the volume increases in LID, we examined the expression of GFAP. As expected, an increased GFAP+ astrocyte was observed in the damaged striatum with 6-OHDA lesions, which were further increased by L-DOPA treatment. This is consistent with previous reports of significant astrogliosis in the lesioned striatum (Bortolanza et al., 2015; Ramírez-García et al., 2015; Fonteles et al., 2020). Contrastingly, another study observed no related differences between rats treated with saline and L-DOPA (Fletcher et al., 2020), arguing against the contribution of astrogliosis to the volume expansion. This discrepancy could be attributed to the administration of higher L-DOPA dose (12 mg/kg, i.p. here) and the use of an alternative delivery route. Given the multiple roles of astrocytes in the nervous system, this discrepancy is not surprising. Astrocytes could be involved in LID development through several processes. First, astrocytes, neurons, and vascular cells, which form the “neurovascular unit,” are crucially involved in regulating local blood flow and blood–brain barrier (BBB) permeability (Yu et al., 2020). Changes in these parameters may alter the kinetics of L-DOPA entering the damaged brain area, and therefore, promote the occurrence of motor complications (Westin et al., 2006). Second, activated astrocytes produce excessive proinflammatory factors (e.g., IL-1β, TNF-α, COX-2, and iNOS), which are associated with LID (Bortolanza et al., 2015, 2021; Dos Santos Pereira et al., 2021). Third, astrocytes are chemically excitable cells that express numerous receptors (including glutamate receptors),induce synapse formation, and secrete gliotransmitters, which allows modulation of synaptic plasticity and neural excitability (Blanco-Suarez et al., 2017). Through these functions, astrocytes influence the activity of cortical basal ganglia networks and contribute to LID development (Obeso et al., 2000). Taken together, our findings suggest that increased GFAP+ astrocyte density may be a good marker for L-DOPA-induced structural plasticity.

Previous studies have elegantly demonstrated that L-DOPA exposure modifies the brain microvasculature. For example, Booth et al. (2021) recently reported that a 10 mg/kg L-DOPA dosing regimen induced significant angiogenesis in the striatum and SN, which was accompanied by a local vasomotor reaction. Similar results have been reported, including increased BBB permeability, blood vessel length, and cerebral blood flow, in L-DOPA-treated dyskinetic rats, which further indicate angiogenesis (Lindgren et al., 2009; Ohlin et al., 2011; Aljuaid et al., 2019). Nevertheless, our team failed to find association of angiogenesis between the diseases and volume changes in both the lesioned and treated groups. Consistent with our finding, Westin et al. (2006) reported no changes in blood vessel length in the striatum; however, it was significantly increased in the entopeduncular nucleus and SN. A recent study on the L-DOPA effects on angiogenesis yielded similar negative results by measuring immunoreactivity for RECA1, which is an anti-endothelial cell antibody and a blood vessel marker (Fletcher et al., 2020). These inconsistent findings could be attributed to differences in the selection of angiogenesis indicators (markers of endothelial proliferation or immature endothelium), L-DOPA dose, and decapitation time. Therefore, there is a need for further studies to confirm whether changes in the striatal microvasculature are related to MRI-derived volume expansion in LID rat models.

A wealth of evidence has shown that LID is related to maladaptive striatal synaptic plasticity (Zhang et al., 2013; Suarez et al., 2016). Since this may be associated with L-DOPA-mediated volume increase, we first measured the levels of several synapse-associated proteins. As key presynaptic and postsynaptic components, respectively, SYP, PSD95, and SAP97 are believed to be involved in synaptic activity (Tarsa and Goda, 2002; Sheng and Kim, 2011). We found that L-DOPA treatment increased above synapse-associated proteins, which is consistent with previous reports that repeated L-DOPA administration increased PSD95 and SAP97 levels (Nash et al., 2005). PSD95 inhibition has been reported to suppress augmented NR2B tyrosine phosphorylation and the interactions of Fyn with NR2B, which facilitate LID management (Ba et al., 2015). Similarly, Porras et al. (2012) reported that PSD95 downregulation or disruption of the interaction between D1R and PSD95 interfered with the establishment of rat and macaque models of AIMs as well as reduced the severity of AIMs. PSD95 or SAP97 overexpression can induce synaptic potentiation, long-term depression, and spinal enlargement (El-Husseini et al., 2000; Rumbaugh et al., 2003; Stein et al., 2003; Nakagawa et al., 2004). Fabrizio et al., found that regulating or destroying the interaction between NMDA receptor NR2A/NR2B and membrane-associated guanylate kinase (MAGUK) family scaffold proteins (including PSD95, SAP97, and SAP102) can alleviate dyskinetic motor behavior (Gardoni, 2006; Gardoni et al., 2012; Mellone et al., 2015). PSD95 and SAP97 can influence each other, which affects postsynaptic density size and spine volume (Cai et al., 2006). There have been no reports of increased SYP in LID. The increased levels of SYP, which is a structural synapse marker, in the lesioned striatum following chronic L-DOPA administration found in this project may be related to new synapse formation, axonal sprouting, and aberrant synaptic plasticity. In summary, we speculated that the L-DOPA-induced upregulation of synapse-associated proteins in the lesioned striatum may be crucially involved in LID development. However, the mechanisms underlying these alterations remain unknown.

Maladaptive synaptic plasticity in the striatum, frontal lobe, and/or thalamus-cortex can cause morphological remodeling (Suarez et al., 2016; Picconi et al., 2018a). Moreover, studies have reported a significant reduction in the dendritic spine density of MSNs expressing D2-receptor (D2R) and/or D1-receptor (D1R) in animals with striatal dopaminergic denervation (Fieblinger et al., 2014; Nishijima et al., 2014; Suárez et al., 2014; Gomez et al., 2019). This is in accordance with a previous report of decreased dendritic spine density in post-mortem striatal tissue obtained from patients with PD compared with healthy controls (Stephens et al., 2005). Moreover, this is consistent with the striatal atrophy in parkinsonian rats observed in our study and several MRI studies on rat and primate models of PD (Westphal et al., 2016; Modo et al., 2017). This dendritic spine loss may be a compensatory change that protects MSNs from overt excitotoxic death in the dopamine-denervated state. Notably, chronic L-DOPA treatment restores the spine density of MSNs expressing D2R (Fieblinger et al., 2014; Suárez et al., 2014; Gomez et al., 2019) and increases the spine size of MSNs expressing D1R (Fieblinger et al., 2014; Nishijima et al., 2014). Moreover, dendritic sprouting of direct pathway MSNs has been described in the striatum of denervated dyskinetic rats (Fieblinger et al., 2018). Similarly, serotonergic mechanisms also play an important role in the appearance of LID (Dupre et al., 2008; Bezard et al., 2013). Previous study by Rylander et al. (2010) provided the first evidence that chronic L-DOPA treatment induced sprouting of striatal 5-HT axon terminals, with an increased incidence of synaptic contacts, and a larger dopamine release. Based on this, Tronci et al. (2017) later found that overexpression of BDNF induced striatal serotonin fiber sprouting and increased the susceptibility to LID. Using electron microscopy techniques, Huang et al. (2018) reported that compared with PD group, the LID group had thicker postsynaptic densities, narrower synaptic clefts, and an increased proportion of perforated striatal synapses. Dendritic spines are the main postsynaptic structures of excitatory synapses and are crucially involved in neuronal connectivity, information storage, and processing; accordingly, their morphological changes are often driven by changes in the activity of synaptic efficacy and are related to numerous disease states (Fortin et al., 2012). Therefore, the observed volume changes after different treatments may reflect morphological changes of dendritic spines in MSNs. To test this hypothesis directly, we performed Golgi-Cox staining. We observed a significant increase in dendritic spines in striatal MSNs following chronic L-DOPA treatment compared with those in the saline-treated PD group. Since previous reports that PSD95 and SAP97 overexpression individually or cooperatively induced a multifold increase in excitatory shaft synapse density and spine enlargement (El-Husseini et al., 2000; Rumbaugh et al., 2003; Poglia et al., 2010). Further, Tarsa and Goda (2002) demonstrated the crucial role of SYP in regulating activity-dependent synapse formation *in vitro*. The increased synapse-associated protein levels in our study were consistent with the L-DOPA-induced increase in dendritic spines in the lesioned striatum, which suggests that increased synapse-associated protein levels may promote the increase of dendritic spines in the lesioned striatum of LID rats.

Taken together, these results suggest that astrocyte activation and overexpression of synapse-associated proteins may be crucially involved in modulating the dendritic spine density, thus affects synaptic transmission and neuronal circuitry, and therefore contributes to LID ontogeny. This also raises the questions of why in PD, dendrites and their dendritic spines are lost in lesioned striatum and why it could be reversed by chronic L-DOPA treatment? What role do astrocytes play in this process? These are obviously interesting goals for future study that possibly involves researches of the molecular mechanisms governing spine formation and regression.

Although our MRI data *in vivo* confirmed a progressive pattern of brain structural changes induced by chronic L-DOPA treatment, it remains unclear whether these morphological changes are a cause or a consequence of LID. Moreover, there is a need to confirm whether the brain structural changes translate to other rodent or non-human primate models of LID. Additionally, an important limitation of our study is that we did not perform linear regression analysis between histological properties and MR-detected volume changes to determine whether the pathologic changes could directly explain the volume expansion. Future studies should address these issues.

In conclusion, our findings demonstrated the utility and access of VBM in whole-brain morphometric assessment of 6-OHDA lesions and L-DOPA-induced changes in rats. We found that in case of dopaminergic degeneration, astrocyte activation, enrichment of synaptic ultrastructure and signaling proteins in the ipsilateral striatum were associated with the structural changes of striatum in LID rats, although it is still unclear whether these are directly involved in the development of LID and lead to the volume expansion. Combining structural MRI with appropriate animal models of LID could help bridge the gap between clinical and preclinical studies. For example, this could allow assessment of disease occurrence or progression and response to therapy based on brain region-specific changes. Moreover, it could facilitate elucidation of cellular mechanisms underlying MRI signal changes in patients with LID. This could allow a better understanding of the adaptive changes in patients with PD receiving L-DOPA replacement therapy and guide therapy and administration.

## Data Availability Statement

The original contributions presented in the study are included in the article/Supplementary Material, further inquiries can be directed to the corresponding author/s.

## Ethics Statement

The animal study was reviewed and approved by the Institutional Animal Care and Use Committee at Tongji Medical College, Huazhong University of Science and Technology, China.

## Author Contributions

XZ, WC, HL, XC, and YaX conceived and designed the study. XZ, WC, YW, WZ, YY, CC, XiaomanY, JW, XiaomeiY, and YuX performed the experiments. XZ, WC, and YW analyzed the data. XZ and WC wrote the manuscript. HL, XC, and YaX revised the manuscript. All authors read and approved the final manuscript.

## Conflict of Interest

The authors declare that the research was conducted in the absence of any commercial or financial relationships that could be construed as a potential conflict of interest.

## Publisher’s Note

All claims expressed in this article are solely those of the authors and do not necessarily represent those of their affiliated organizations, or those of the publisher, the editors and the reviewers. Any product that may be evaluated in this article, or claim that may be made by its manufacturer, is not guaranteed or endorsed by the publisher.

## Footnotes

1
https://www.fil.ion.ucl.ac.uk/spm/software/spm12/

## Supplementary Material

Supplementary Figure 2 Behavioral differences between day 13 and day 30 after L-DOPA/saline administration. No significant behavioral difference was observed in each group over time. **(A)** Adjusting step test. **(B,C)** Coat hanger experiment. **(D,E)** Cylinder test. *N* = 12 (sham); *N* = 11 (PD); *N* = 11 (LID).
