# Supplementary figures and images for: Histological Correlates of Neuroanatomical Changes in a Rat Model of Levodopa-Induced Dyskinesia Based on Voxel-Based Morphometry
Source: Front Aging Neurosci. 2021 Oct 28;13:759934. doi: 10.3389/fnagi.2021.759934 (PMC8581620; doi:10.3389/fnagi.2021.759934)

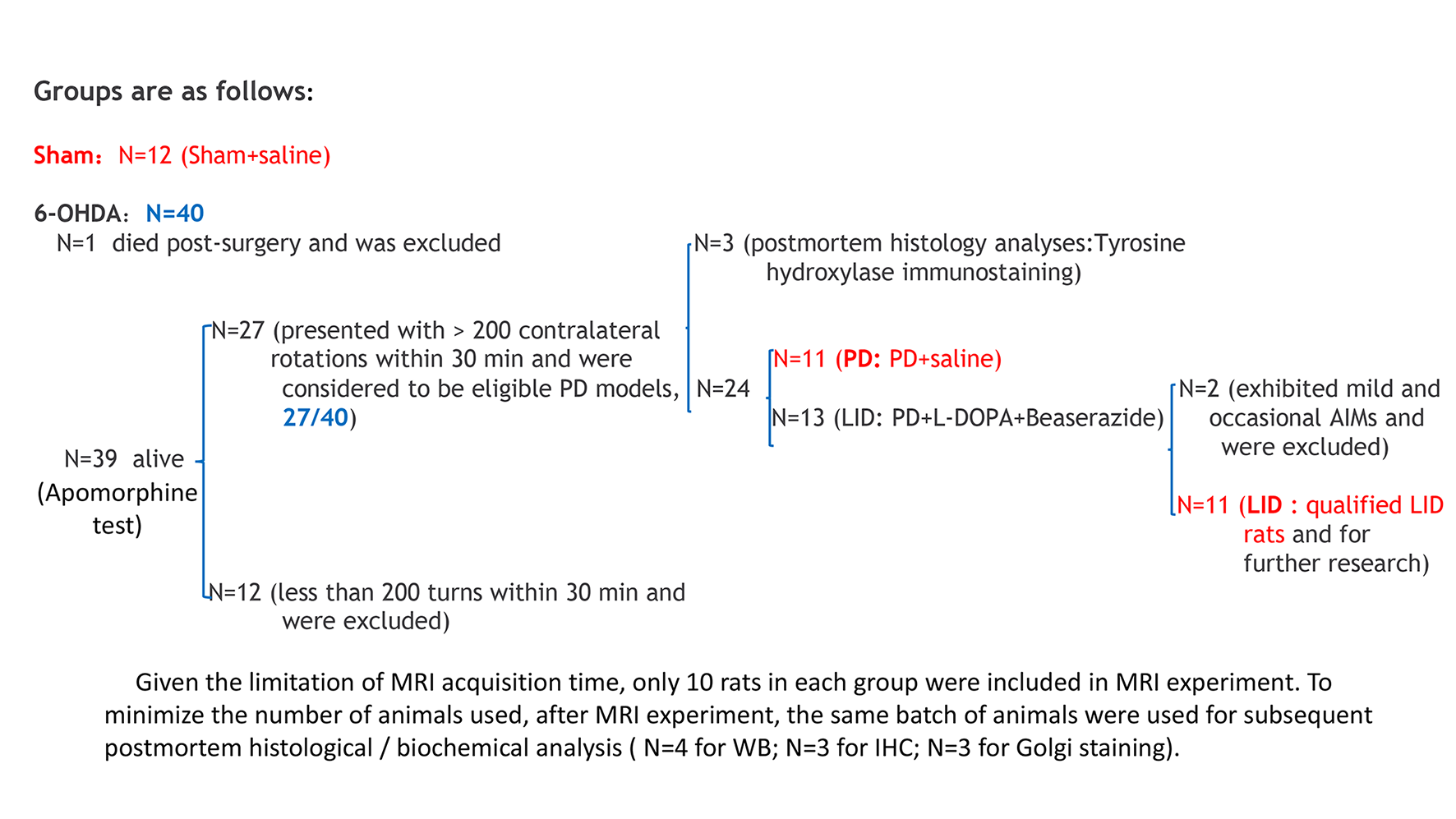

Supplement: Supplementary file 1 [file Image_1.tif]

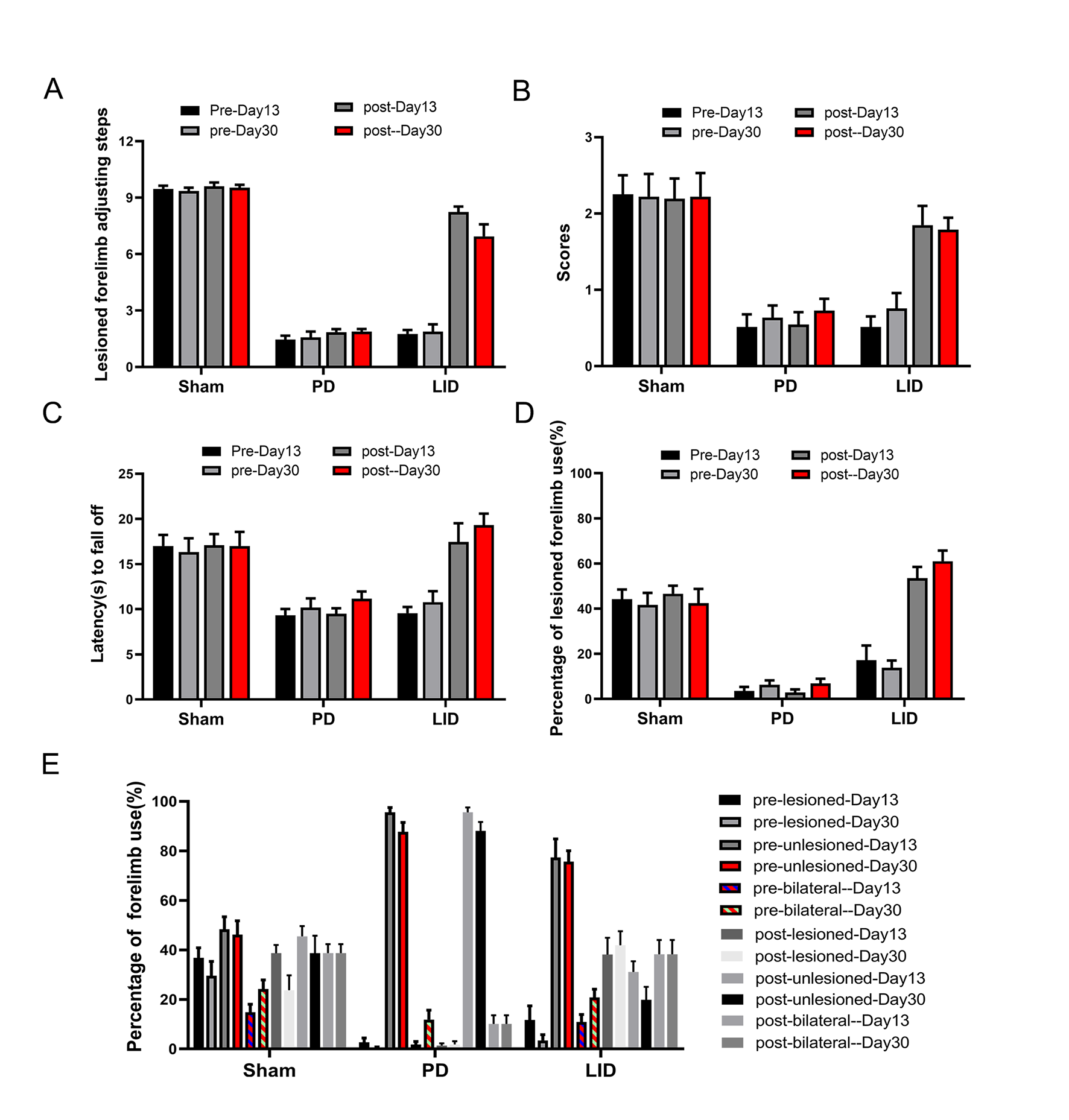

Supplement: Supplementary Figure 2 — Behavioral differences between day 13 and day 30 after L-DOPA/saline administration. No significant behavioral difference was observed in each group over time. (A) Adjusting step test. (B,C) Coat hanger experiment. (D,E) Cylinder test. N = 12 (sham); N = 11 (PD); N = 11 (LID). [file Image_2.tif]
