# Supplementary material for: Histological Correlates of Neuroanatomical Changes in a Rat Model of Levodopa-Induced Dyskinesia Based on Voxel-Based Morphometry
Source: Front Aging Neurosci. 2021 Oct 28;13:759934. doi: 10.3389/fnagi.2021.759934 (PMC8581620; doi:10.3389/fnagi.2021.759934)
Supplement: Supplementary file 3 [file Table_1.docx]

**Supplementary Table 1. Statistical comparison of the volume in regional gray matter with two sample t-test at 2weeks and 1month.** MRI images were first segmented into gray matter, white matter and cerebrospinal fluid based on probability template which created in our lab. And we can differentiate each brain region by the template, and the volume were extracted and calculate by matlab. Voxels thresholded at P< 0.001, uncorrected. H, hemisphere; L, contralateral unlesioned hemisphere; R, ipsilateral 6-OHDA lesioned hemisphere; B, both hemispheres. Volumes were presented as mean ± SD (N = 10/group).

| Brain region | H | Time point |  |  | Volume /mm^3^ |  |  |  | P value |  |
| --- | --- | --- | --- | --- | --- | --- | --- | --- | --- | --- |
|  |  |  |  | Sham | PD | LID |  | PD vs. Sham | LID vs. PD | LID vs. sham |
| Cingulate cortex, area 1 | B | 2 weeks |  | 1.16±0.08 | 1.12±0.07 | 1.16±0.12 |  | 0.179 | 0.323 | 0.945 |
|  |  | 1 month |  | 1.17±0.07 | 1.04±0.06 | 1.08±0.10 |  | < 0.001 | 0.250 | 0.051 |
| Cingulate cortex, area 2 | B | 2 weeks |  | 3.73±0.20 | 3.55±0.22 | 3.65±0.36 |  | 0.079 | 0.454 | 0.553 |
|  |  | 1 month |  | 3.72±0.14 | 3.30±0.19 | 3.44±0.31 |  | < 0.001 | 0.267 | 0.016 |
| Substantia nigra | R | 2 weeks |  | 0.31±0.02 | 0.25±0.06 | 0.38±0.06 |  | 0.002 | < 0.001 | 0.003 |
|  |  | 1 month |  | 0.31±0.03 | 0.17±0.05 | 0.31±0.07 |  | < 0.001 | < 0.001 | 0.852 |
| Field CA1 of  hippocampus | L | 2 weeks |  | 5.29±0.41 | 4.57±0.90 | 4.78±0.74 |  | 0.011 | 0.513 | 0.074 |
|  |  | 1 month |  | 5.16±0.35 | 4.21±0.36 | 4.55±0.71 |  | < 0.001 | 0.197 | 0.03 |
| Striatum | R | 2 weeks |  | 10.98±0.34 | 10.45±0.80 | 12.12±1.67 |  | 0.076 | 0.010 | 0.048 |
|  |  | 1 month |  | 11.05±0.39 | 9.42±1.09 | 11.67±1.82 |  | < 0.001 | < 0.001 | 0.308 |
| Piriform cortex | R | 2 weeks |  | 3.59±0.18 | 3.26±0.14 | 3.40±0.37 |  | < 0.001 | 0.287 | 0.156 |
|  |  | 1 month |  | 3.50±0.15 | 2.87±0.20 | 3.15±0.47 |  | < 0.001 | 0.095 | 0.037 |
| Olfactory bulb | B | 2 weeks |  | 8.56±1.53 | 8.92±0.82 | 7.27±1.15 |  | 0.519 | 0.002 | 0.048 |
|  |  | 1 month |  | 6.52±1.10 | 5.04±0.52 | 4.73±0.53 |  | 0.001 | 0.209 | < 0.001 |
| Ectorhinal cortex | R | 2 weeks |  | 1.86±0.12 | 1.62±0.16 | 1.65±0.19 |  | 0.002 | 0.659 | 0.010 |
|  |  | 1 month |  | 1.81±0.14 | 1.47±0.16 | 1.59±0.22 |  | < 0.001 | 0.191 | 0.019 |
| Primary visual cortex | R | 2 weeks |  | 1.11±0.07 | 1.04±0.11 | 1.08±0.14 |  | 0.089 | 0.510 | 0.469 |
|  |  | 1 month |  | 1.06±0.03 | 0.91±0.07 | 0.99±0.14 |  | < 0.001 | 0.134 | 0.137 |
| Secondary motor cortex | B | 2 weeks |  | 15.31±0.96 | 14.42±0.57 | 15.15±1.38 |  | 0.022 | 0.116 | 0.762 |
|  |  | 1 month |  | 15.19±0.63 | 13.89±0.50 | 14.50±1.36 |  | < 0.001 | 0.200 | 0.166 |
